# Biomarker analysis of the phase 3 TORCH trial for first line erlotinib *versus* chemotherapy in advanced non-small cell lung cancer patients

**DOI:** 10.18632/oncotarget.15725

**Published:** 2017-02-25

**Authors:** Lucia Kim, Mauro Saieg, Massimo Di Maio, Ciro Gallo, Charles Butts, Fortunato Ciardiello, Ronald Feld, Dengxiao Cheng, Vittorio Gebbia, Marco Angelo Burgio, Yasmin Alam, Simona Signoriello, Antonio Rossi, Natasha Leighl, Paolo Maione, Alessandro Morabito, Geoffrey Liu, Ming-Sound Tsao, Francesco Perrone, Cesare Gridelli

**Affiliations:** ^1^ Department of Pathology, University Health Network, Princess Margaret Cancer Center and Department of Laboratory Medicine and Pathobiology, University of Toronto, Toronto, Canada; ^2^ National Cancer Institute, G. Pascale Foundation, IRCCS, Naples, Italy; ^3^ Department of Mental Health and Preventive Medicine, Chair of Statistics, Second University of Naples, Naples, Italy; ^4^ Medical Oncology, Cross Cancer Institute, Edmonton, Canada; ^5^ Medical Oncology, Second University, Naples, Italy; ^6^ Division of Hematology and Oncology, Princess Margaret Cancer Centre and Department of Medicine, University of Toronto, Toronto, Canada; ^7^ Medical Oncology, Casa di Cura La Maddalena, Palermo, Italy; ^8^ Department of Medical Oncology, Istituto Scientifico Romagnolo per lo Studio e la Cura dei Tumori IRCCS, Meldola, Italy; ^9^ Medical Oncology, Windsor Regional Cancer Centre, Windsor, Canada; ^10^ Department of Oncology/Hematology, Division of Medical Oncology, “S.G. Moscati” Hospital, Avellino, Italy; ^11^ On behalf of the TORCH Investigators; ^12^ Department of Pathology, Inha University School of Medicine, Incheon, South Korea; ^13^ Santa Casa Medical School, Sao Paulo, SP, Brazil; ^14^ University of Turin, Turin, Italy

**Keywords:** NSCLC, EGFR TKI, biomarker analysis, predictive factors, prognostic factors

## Abstract

**Background:**

The TORCH phase III trial compared the efficacy of first-line erlotinib followed by chemotherapy at progression (experimental arm) with the reverse sequence (standard arm) in unselected advanced non-small cell lung cancer (NSCLC) patients. Here we report biomarker analyses.

**Methods:**

*EGFR* and *KRAS* mutation, expression of EGFR family members and of cMET and PTEN and *EGFR* and *ABCG2* germline polymorphisms were tested on tumor tissue or blood samples to either confirm previously proposed predictive role or describe it in an explorative setting. Progression-free survival (PFS) was the primary end-point, overall survival, response rate and side effects (diarrhoea and skin toxicity) were secondary end-points. Interactions between biomarkers and treatment were studied with multivariable models (either Cox model or logistic regression). Statistical analyses accounted for multiple comparisons.

**Results:**

At least one biomarker was assessed in 324 out of 760 patients in the TORCH study. *EGFR* mutation was more common in female (*P* = 0.0001), East Asians (*P* < 0.0001) and never smoker (*P* < 0.0001) patients; low MET protein expression by IHC (H-score <200) was more frequent in squamous (*P* < 0.00009) and ABCG2 C/A or A/A polymorphism was more frequent among East-Asian patients (*P* = 0.0003). A significant interaction was found for *EGFR* mutation in PFS and response rate analyses while no predictive effect on OS was found for any biomarker. No biomarker tested was prognostic for PFS and OS. No polymorphism was significantly associated with skin toxicity or diarrhea.

**Conclusion:**

In the present study, beyond the known role of *EGFR* mutation, no other biomarker has predictive or prognostic role.

## INTRODUCTION

The discovery of oncogenic mutations in the tyrosine kinase (TK) domain of the epidermal growth factor receptor (*EGFR*) gene, and sensitivity of mutant lung cancers to EGFR-TK inhibitors (TKIs), have revolutionized the treatment of advanced NSCLC. Erlotinib, one of the first generation EGFR-TKIs, has become a standard first line drug for patients with *EGFR* mutant lung cancers.[1] However, only 60-80% of NSCLC patients with *EGFR* mutant tumour respond to EGFR-TKI therapy,[2–5] while a small proportion of patients with *EGFR* wild-type tumors may also benefit from this class of drugs.[6, 7] Primary resistance to EGFR-TKIs has been attributed to various factors, including *EGFR* exon 20 insertion mutations.[8, 9] Patients whose tumors harbor *KRAS* mutation are rarely responsive to EGFR-TKIs and *KRAS* mutation might serve as a predictor of resistance to EGFR-TKIs.[10, 11] Activation of alternative signaling pathways including mutations in *BRAF, PIK3CA* and loss of PTEN have also been implicated as resistance mechanisms in preclinical studies.[12, 13] In addition, germline polymorphisms involving the promoter and intron 1 transcription enhancer regions of the *EGFR* gene and the *ABCG2* multidrug transporter gene have also been reported as modifiers of response to EGFR-TKI therapy. [14–16]

The TORCH (Tarceva OR CHemotherapy) trial was an Italian-Canadian multicenter, open-label, randomized phase III trial comparing first line erlotinib followed by chemotherapy (cisplatin-gemcitabine) at progression, with the reverse standard sequence of first-line chemotherapy followed by erlotinib, in unselected advanced stage IIIB and IV, predominantly Caucasian, NSCLC patients.[17] The study was terminated early due to inferiority of the experimental arm (erlotinib first) in terms of overall survival (OS). In this manuscript, we summarize the results of confirmatory and exploratory analyses of the impact of biomarkers on clinical outcomes in this trial including *EGFR* gene copy number gains, *KRAS* mutations, immunohistochemical expression of EGFR family members, cMET and PTEN, and *EGFR* and *ABCG2* germline polymorphisms, in addition to *EGFR* mutations that have already been partially reported.[17]

## RESULTS

Details of patients’ flow and samples available for each biomarker are reported in Supplementary Figure S2. 556 patients consented to biomarker studies and at least one biomarker was tested for 324 (42.6%) patients (study population). Baseline characteristics of the biomarker population were comparable to both the population of patients (*N* = 673) enrolled in centers that provided at least one sample and the whole trial patient population (*N* = 760) (Supplementary Table S2).

Distribution of biomarkers categories is reported in Table 1. All biomarkers were balanced between treatment arms. Due to low prevalence or absence of positive cases, HER2 and HER3 were excluded from further analyses.

**Table 1:** Distribution of biomarkers according to treatment arm, within the biomarker population (n=324 patients with at least one biomarker available)

Biomarker	Category	Standard Arm (G/C) (*N*=164)	Experimental Arm (E) *(N=160)*	Overall (*N*=324)	*P* value*
***Genomic Testing***					
*KRAS* mutation	Wild type	101 (62%)	102 (64%)	203 (63%)	0.49
	Mutant	35 (21%)	38 (24%)	73 (23%)	
	Unknown	28 (17%)	20 (12%)	48 (15%)	
*EGFR* mutation	Wild type	116 (71%)	120 (75%)	236 (73%)	0.59
	Mutant	20 (12%)	19 (12%)	39 (12%)	
	Unknown	28 (17%)	21 (13%)	49 (15%)	
*EGFR* gene copy	Low	41 (25%)	53 (33%)	94 (29%)	0.27
	High	54 (33%)	48 (30%)	102 (31%)	
	Unknown	69 (42%)	59 (37%)	128 (40%)	
***Immunohistochemical Staining***					
EGFR IHC score	Low	76 (46%)	71 (44%)	147 (45%)	0.71
	High	7 (4%)	10 (6%)	17 (5%)	
	Unknown	81 (49%)	79 (49%)	160 (49%)	
PTEN	Negative	17 (10%)	13 (8%)	30 (9%)	0.70
	Positive	57 (35%)	61 (38%)	118 (36%)	
	Unknown	90 (55%)	86 (54%)	176 (54%)	
MET-H200	Low	40 (24%)	45 (28%)	85 (26%)	0.75
	High	36 (22%)	33 (21%)	69 (21%)	
	Unknown	88 (54%)	82 (51%)	170 (52%)	
MET-IHC	Negative	32 (20%)	32 (20%)	64 (20%)	0.90
	Positive	44 (27%)	46 (29%)	90 (28%)	
	Unknown	88 (54%)	82 (51%)	170 (52%)	
HER2	Low	59 (36%)	56 (35%)	115 (35%)	0.83
	High	2 (1%)	1 (1%)	3 (1%)	
	Unknown	103 (63%)	103 (64%)	206 (64%)	
HER3	Low	74 (45%)	70 (44%)	144 (44%)	0.80
	High	0	0	0	
	Unknown	90 (55%)	90 (56%)	180 (56%)	
*Germline polymorphisms*					
*EGFR*-216	G/G	38 (23%)	40 (25%)	78 (24%)	0.52
	G/T or T/T	79 (48%)	83 (52%)	162 (50%)	
	Unknown	47 (29%)	37 (23%)	84 (26%)	
*EGFR*-191	C/C	95 (58%)	100 (62%)	195 (60%)	0.52
	C/A or A/A	22 (13%)	23 (14%)	45 (14%)	
	Unknown	47 (29%)	37 (23%)	84 (26%)	
*EGFR* CA repeat	S/S	29 (18%)	45 (28%)	74 (23%)	0.06
	S/L or L/L	99 (60%)	89 (56%)	188 (58%)	
	Unknown	36 (22%)	26 (16%)	62 (19%)	
*ABCG2*	*C/C*	104 (63%)	114 (71%)	218 (67%)	0.32
	*C/A* or *A/A*	22 (13%)	17 (11%)	39 (12%)	
	Unknown	38 (23%)	29 (18%)	67 (21%)	

*Chi square test, comparing Standard and Experimental Arms; *EGFR CA* repeat S (short): ≤16CA repeat; L (Long): ≥17CA repeat; Unknowns are due to lack of remaining material available for biomarker assessment.

Associations between each biomarker and baseline patients characteristics are described in Supplementary Tables S3 to S13. According to the predefined 0.001 threshold for statistical significance, *EGFR* mutation was significantly more common in female (*p* = 0.0001), East Asians (*P* < 0.0001) and never smoker (*P* < 0.0001) patients; low MET protein expression by IHC (H-score < 200) was more frequent in case of squamous carcinoma (*P* = 0.00009) and ABCG2 C/A or A/A polymorphism was more frequent among East-Asian patients (*P* = 0.0003).

Pairwise association between biomarkers is reported in Supplementary Table S14. No association was significant at the predefined level of 0.001, with the exception of the obvious one between the two scoring systems for MET protein expression by IHC.

**Figure 1:**
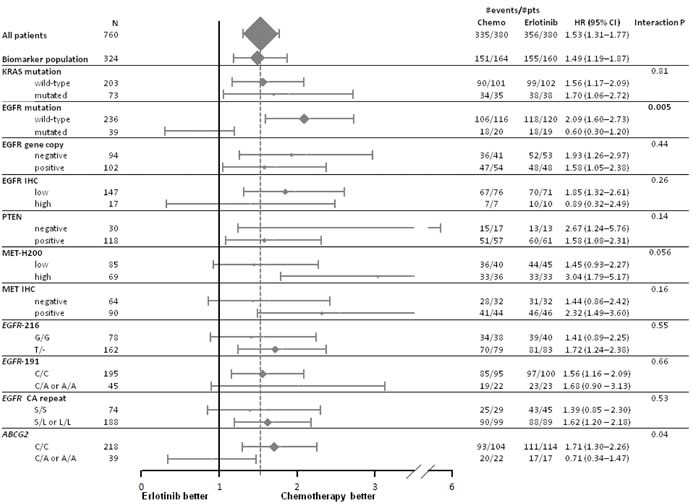
Forest plot of progression free survival by treatment arm and biomarkers Hazard ratio (HR) < 1 means a lower risk of progression or death for patients treated with first-line erlotinib.

**Figure 2:**
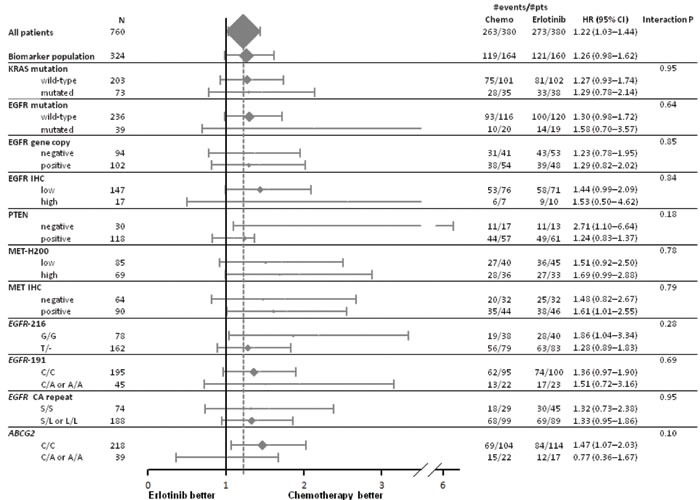
Forest plot of overall survival by treatment arm and biomarkers Hazard ratio (HR) < 1 means a lower risk of death for patients treated with first-line erlotinib.

**Figure 3:**
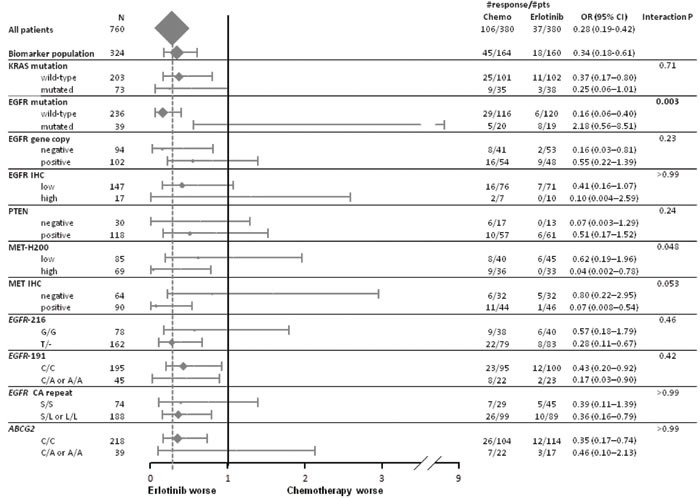
Forest plot of response by treatment arm and biomarkers Odds ratio (OR) >1 means a higher probability of response for patients treated with first-line erlotinib.

The modifying effects of biomarkers on PFS, OS and response to first treatment are provided in Figures 1 to 3. A significant interaction was only found for *EGFR* mutation in PFS (Figure 1) and response rate analyses (Figure 3) while no predictive effect on OS was found for any biomarker (Figure 2) at the predefined Bonferroni-Holm sequential significance levels.

In an exploratory unplanned analysis of patients with non-mutated *EGFR* or *EGFR* mutation unknown status, ABCG2 polymorphism had a significant (*P* = 0.003) interaction with treatment effect on PFS (HR for erlotinib vs chemotherapy was 0.62, 95%CI 0.28-1.34 among A/C or A/A and 2.07, 95%CI 1.54-2.80 among C/C ABCG2 polymorphisms). Further, adjustment for the Asian ethnicity, which was significantly more common in the *A/C* or *A/A* arm when compared to the *C/C* arm, led to similar results.

None of the tested biomarkers was prognostic for PFS and OS at the pre-defined significance level of 0.01 (Table 2).

**Table 2:** Prognostic role of biomarkers with no significant interaction with treatment

	Progression-free survival			Overall survival	
	Hazard ratio (95% CI)	*P* value		Hazard ratio (95% CI)	*P* value
***EGFR* gene copy**					
High vs low	0.82 (0.61 – 1.10)	0.19		0.77 (0.56 – 1.06)	0.11
**EGFR IHC**					
High vs low	1.06 (0.64 – 1.77)	0.82		1.00 (0.58 – 1.72)	0.99
***KRAS* mutation**					
Mutant vs wild type	1.23 (0.93 – 1.61)	0.14		1.13 (0.84 – 1.52)	0.42
**PTEN**					
Positive vs negative	0.99 (0.65 – 1.51)	0.95		1.13 (0.71 – 1.81)	0.61
**MET-H200**					
High vs low	1.39 (0.99 – 1.95)	0.055		1.42 (0.99 – 2.05)	0.059
**MET-IHC**					
Positive vs negative	1.50 (1.07 – 2.10)	0.02		1.46 (1.01 – 2.13)	0.047
***EGFR* SNP 216**					
G/G vs T/-	0.78 (0.59 – 1.03)	0.082		0.67 (0.48 - 0.94)	0.019
***EGFR* SNP 191**					
C/C vs A/-	1.13 (0.80 – 1.58)	0.49		1.19 (0.80 – 1.78)	0.38
***ABCG2***					
C/C vs A/-	1.10 (0.78 – 1.57)	0.58		1.10 (0.73 – 1.66)	0.66
***EGFR* CA repeat**					
S/S vs L/-	0.94 (0.71 – 1.25)	0.67		0.89 (0.64 – 1.24)	0.48

None of the polymorphisms evaluated were significantly associated with skin toxicity or diarrhea (Supplementary Table S15).

## DISCUSSION

As an ancillary analysis of the TORCH trial, we explored potential biomarkers for predicting the response or resistance to the EGFR-TKIs compared with chemotherapy. We assessed both biomarker-by-treatment interactions and prognostic value of the proposed biomarkers. Follow-up data were mature and a conservative statistical analysis plan was applied accounting for multiplicity of comparisons and reducing the expected inflation of false positive results. As a major limitation, however, biomarkers’ evaluation was not mandatory in the trial and was actually performed only in 43% of the patients; nonetheless, this rate compares well with other trials in advanced lung cancer where biologic sample collection was not mandatory.

Except for *EGFR* mutation,[17] we failed to confirm the predictive role of any other biomarker, including high expression of HER2, HER3, cMET and loss of PTEN expression, that were studied due to their potential role in activating bypass survival pathway downstream of EGFR.[18, 19] Among these markers, we found no or very few cases with high expression of HER2 and HER3. Interestingly, the first treatment progression free hazard ratio for high and low cMET expression (H-score ≥200 vs < 200) was 3.04 vs. 1.45, with borderline interaction p value of 0.056. These results confirm that first line chemotherapy is superior to erlotinib irrespective of cMET expression level, however suggesting that any potential activity of erlotinib is significantly less in tumor that express very high level of cMET. Unfortunately, the low number of patients with *EGFR* mutant tumor exclude us from studying potential role of high MET expression as a potential negative predictive marker in first line EGFR TKI therapy.

The predictive role of *EGFR* gene copy number as determined by fluorescence in situ hybridization (FISH) is a matter of controversy.[3, 20–22] As high percentages of *EGFR* mutant NSCLC also demonstrate amplification of the gene, it was postulated that the predictiveness of *EGFR* high gene copy number could be accounted by the presence of mutation. In fact, in the IPASS patients, EGFR FISH was not predictive in *EGFR* wild type patients.[23] The results of our analysis in the TORCH patients confirm this finding (Supplementary Figure S3).

Despite suggestions that germline polymorphisms may be associated with erlotinib efficacy, none has consistently been associated with survival or response.[15, 16] In the TORCH trial, we found a significant interaction between the *ABCG2 +421* polymorphism and PFS. The non significant finding of interaction for OS (*P* = 0.11) may have been favoured by the cross-over design. In this trial, the vast majority of patients likely carried wild type *EGFR*, which is the primary driver of best initial response. In contrast, wild type *EGFR* produces mostly stable disease responses, and thus it was unlikely that initial response would be differentiated in this population by genetic polymorphisms; indeed we found no relationship between any of the polymorphisms and best initial response to therapy. Two exploratory analyses of *ABCG2 +421* were performed. This *ABCG2* polymorphism had been evaluated in one other randomized control trial involving erlotinib as the experimental drug but in a different setting: the BR.21 trial of chemo-refractory placebo-controlled metastatic/advanced stage patients found no significantly interaction associations between this polymorphism and the trial arm for any treatment outcomes.[15] The same polymorphisms may affect toxicity.[15, 24] However, in the TORCH trial, we found no significant or even trends in relationship with any of the main toxicities of erlotinib, but small samples sizes limited any firm conclusions.

In conclusion, an extensive exploratory biomarker analysis in TORCH trial failed to identify additional predictive or prognostic biomarkers beyond *EGFR* mutation in first line erlotinib therapy.

## MATERIALS AND METHODS

The details of the TORCH clinical trial eligibility and conduct have been described previously.[17]

Formalin fixed paraffin embedded blocks or unstained sections of tumor tissue were collected. Because of limited tissue materials, biomarker analyses were performed in the following priority order: *EGFR* mutation status, *KRAS* mutation, *EGFR* gene copy number (GCN) by fluorescent in situ hybridization (FISH), EGFR, cMET, HER2, HER3 and PTEN protein expression by immunohistochemistry. The remaining material was used for genotyping of *EGFR* and *ABCG2* polymorphisms (when no blood sample or tissue block of normal tissue was available). No prioritization was required for blood sample derived DNA.

All biomarker assays were performed at the Applied Molecular Profiling Laboratory at the Princess Margaret Cancer Centre. All tissue analyses were preceded by a pathology review of the hematoxylin eosin (HE) stained slides to confirm the diagnosis, estimate the tumor cell abundance and mark the tumor areas for macrodissection or FISH scoring. The procedure of the *EGFR* mutation test and *EGFR/ABCG2* polymorphism analysis has been described previously.[15, 17, 25]

*EGFR* exon 19 deletion and exon 21 L858R mutations were analysed as previously reported.[17] *KRAS* codon 12 and 13 mutations were analysed using the capilllary sequencing method, with positive or equivocal results confirmed by repeat sequencing. *EGFR* gene copy number was evaluated by fluorescence in situ hybridization; high and low copy number cases were categorized using the University of Colorado system, as reported previously.[25]

IHC staining was performed using the BenchMark XT autostainer (Ventana, Tucson, AZ). The antibodies, staining conditions and scoring cut-offs used are detailed in Supplementary Table S1. For each marker, the staining intensity (grades 0 to 3) and estimated percentage (%) of tumor cells at each intensity grade were recorded. The H-score was the sum of % stained tumor cells at each staining intensity. For EGFR, HER2 and HER3, cases were classified as low or high expression, using H-score < 200 and ≥200, respectively. Two scoring systems were used for cMET: (1) MET-H200 scoring system using H-score < 200 and ≥200 as above; and (2) the MET IHC scoring system that was developed in a previous trial.[26] The latter score was assessed independently by two pathologists (LK and MST) and the final MET-IHC represents the mean of the two independent scores (Supplementary Figure S1). PTEN IHC was interpreted as negative when there was no staining at all.[27] All interpretation was performed without information on clinical outcome and assigned treatment strategy.

Blood samples were collected using a kit provided by the central laboratory with specific processing and shipment instructions. DNA was isolated using the Qiagen humanDNA kit. Polymorphism analyses for *EGFR* promoter *(-216 G>T*, rs712829 and *-191 C>A* rs712830), *EGFR* intron 1*CA* repeat polymorphisms and for the *ABCG2 +421 C>A* (rs2231142) polymorphisms have been described previously.[15, 16] In brief, analyses were conducted using direct Sanger sequencing and Taqman polymerase chain reaction (PCR) reaction. For patients without blood samples, analyses were conducted using DNA isolated from FFPE tumor samples used for mutation analyses, as a surrogate material. Our prior work has shown this region to be highly preserved when comparing germline to somatic material from lung cancer, with >90% agreement in paired blood-tumor tissue analyses.[15, 16]

The statistical analysis plan is detailed in the Supplementary materials on line. Several biomarkers were evaluated in confirmatory (*EGFR* mutation, *EGFR* gene copy, EGFR IHC, *KRAS* mutation, polymorphic variants of *EGFR* (SNP 216, SNP 191, CA repeat) and ABCG2) and exploratory (PTEN, MET.H200, MET IHC, HER2, HER3) analyses. Because of multiplicity of comparisons different significance levels were used according to a pre-defined step-down Holm-Bonferroni sequential testing procedure.

All efficacy analyses were based on intention-to-treat strategy. The primary end-point was progression-free survival (PFS) to first treatment, while overall survival (OS) and response rate to first treatment (RR) were secondary end-points.

Both predictive and prognostic roles of biomarkers were evaluated using the appropriate multivariable models.

## SUPPLEMENTARY MATERIALS FIGURES AND TABLES


