# Supplementary material for: Biomarker analysis of the phase 3 TORCH trial for first line erlotinib versus chemotherapy in advanced non-small cell lung cancer patients
Source: Oncotarget. 2017 Feb 25;8(34):57528–36. doi: 10.18632/oncotarget.15725 (PMC5593664; doi:10.18632/oncotarget.15725)
Supplement: Supplementary file 1 [file oncotarget-08-57528-s001.pdf]

Biomarker analysis of the phase 3 TORCH trial for first line erlotinib *versus* chemotherapy in advanced non-small cell lung cancer patients

Supplementary Material

Summary

Statistical analysis plan 2
Table S1. Antibodies used for immunohistochemistry studies. 4
Table S2. Baseline characteristics of patients 5
Table S3. Patients characteristics by EGFR mutational status 6
Table S4. Patients characteristics according to EGFR gene copy number 7
Table S5. Patients characteristics according to KRAS mutational status 8
Table S6. Patients characteristics according to EGFR IHC 9
Table S7. Patients characteristics according to PTEN IHC 10
Table S8. Patients characteristics according to c-MET IHC H-score (low vs high) 11
Table S9. Patients characteristics according to c-MET IHC modified score 12
Table S10. Patients characteristics according to EGFR -216 genotype category 13
Table S11. Patients characteristics according to EGFR -191 genotype category 14
Table S12. Patients characteristics according to ABCG2 +421 polymophism 15
Table S13. Patients characteristics according to EGFR CA dinucleotide repeat length genotypes 16
Table S14. Relationships between biomarkers expressed as odds ratios 17
Table S15. Skin toxicity and diarrhea according to polymorphisms 18
Figure S1. Comparison of the 2 independent cMET H-scores. .... **Error! Bookmark not defined.**
Figure S2. Patient study flow and conduction of biomarker study by treatment arm .... **Error! Bookmark not defined.**
Figure S3. Forest plot of HR of progression by *EGFR* gene copy number in *EGFR* wild type patients 21
TORCH Investigators 22

Statistical analysis plan

In this ancillary study of the TORCH trial several biomarkers were evaluated, thus statistical analyses had to take into account the multiplicity of comparisons and to reduce the expected inflation of false positive results. As for tumor-based biomarkers, the analysis was confirmatory (detailed in the protocol) for some biomarkers (*EGFR* mutation, *EGFR* gene copy, EGFR IHC and *KRAS* mutation), while it was exploratory (not detailed in the protocol) for the other biomarkers (PTEN, MET.H200, MET IHC, HER2, HER3). As for genetic polymorphisms, the analysis was confirmatory (detailed in the protocol) for polymorphic variants of *EGFR* (SNP 216, SNP 191, CA repeat) and ABCG2.

Pre-defined strategy for statistical analysis mainly implied to adopt different significance levels within the three 'families' of biomarkers above, in order to preserve a family-wise error rate α of 0.05. More stringent significant levels were adopted to correct for multiple comparisons with reference to the specific aims of statistical analysis. For all biomarkers, missing values are reported in the descriptive tables but no longer included in statistical analyses.

Association of all biomarkers with patient's baseline characteristics was tested using a pre-defined significance level of 0.001. Pairwise association between biomarkers was tested using a pre-defined significance level of 0.001 and measured by odds ratios.

Efficacy analysis was based on intention-to-treat strategy. The primary end-point was progression-free survival (PFS) to first treatment, while overall survival (OS) and response rate to first treatment (RR) were secondary end-points.

For all biomarkers the main goal was to assess interaction with treatment.

The modifying effect in terms of PFS and OS of each biomarker on treatment arm was assessed by statistical significance of the interaction term biomarker*treatment within a multivariable Cox' model with treatment and biomarker as covariates. Findings for subgroups were reported as Hazard ratios (HR) and 95% confidence intervals (CI). The modifying effect of biomarkers on response rate was assessed by the Zelen exact test for homogeneity of odds ratios of response. Odds ratios (OR) and 95% CI of response rates were estimated in 2x2 contingency tables for each biomarker, stratifying by treatment arm., The analysis of the role of *EGFR* mutation has already been reported in the article reporting the final analysis of the study (Gridelli et al JCo 2012), but for a complete and clearer interpretation of results it is also reported in this biomarker paper.

For every efficacy end-point, the step-down Holm-Bonferroni sequential testing procedure was used to adjust for multiple comparisons when testing interactions using a predefined family-wise error rate α of 0.05. Three families of biomarkers were defined, as detailed above: (i) tumor-based biomarkers, confirmatory analysis (4 biomarkers: *EGFR* mutation, *EGFR* gene copy, EGFR IHC and *KRAS* mutation); (ii) tumor-based biomarkers, exploratory analysis (3 biomarkers: PTEN, MET-H200 and MET IHC, following the exclusion of HER2 and HER3); (iii) genetic polymorphisms (4 biomarkers: EGFR-216, EGFR-191, EGFR CA repeat, ABCG2). Therefore, the four significance levels for sequential testing in the two families with four biomarkers were equal to 0.0125, 0.0167, 0.025, 0.05, while the three sequential significance levels for the family with three biomarkers were equal to 0.0167, 0.025, 0.05.

If no predictive value was found (i.e. if biomarker*treatment interaction was not statistically significant at the above defined levels), the prognostic role of the biomarker was investigated using a pre-defined significance level of 0.01.

Skin toxicity and diarrhea were also investigated for their relationship with genetic polymorphisms only, Both outcomes were dichotomized (any grade of toxicity vs. no toxicity). Only first line of treatment was considered. Similarly to response rate analysis, odds ratios (and 95% CI) were estimated in 2x2 contingency tables for each genetic polymorphism, stratifying by treatment. The modifying effect on toxicity of polymorphisms on treatment arm was assessed by statistical significance of the Zelen exact test for homogeneity of odds ratios.

Supplementary Table S1: Antibodies used for immunohistochemistry studies.

Marker	Antibody clone (Source)	Dilution	Antigen retrieval	Incubation time	Scoring cut-off (Low/Negative vs. High/positive)
EGFR	31G7 (Zymed)	1:100	Protease1 4min	60min	H-score < *vs.* ≥ 200
HER2	4B5 (Ventana)	dispenser	CC1* mild	20min	H-score < *vs.* ≥ 200
HER3	SP71 (Spring BioScience)	1:50	CC1 standard	60min	H-score < *vs.* ≥ 200
cMET	SP44 (Ventana, Tucson, AZ)	dispenser	CC1 standard	32min	MET-IHC score**: 0/1+ *vs.* 2+/3+
					MET-H200: H-score < *vs.* ≥ 200
PTEN	138G6 (Cell Signaling)	1:100	CC1 standard	60min	Negative vs any positive***

*CC1:Tris/Borate/EDTA buffer pH8-8.5

**MET-IHC score was defined in the MetMAb/Onartuzumab trial (Spigel DR, et al. Clin Oncol. 2013 Nov 10;31(32):4105-14) as: 3+ (≥ 50% of tumor cells staining with strong intensity); 2+ (≥ 50% of tumor cells with moderate or higher staining but <50% with strong intensity); 1+(≥50% of tumor cells with weak or higher staining but <50% with moderate or higher intensity); or 0 (no staining or <50%of tumor cells with any intensity).

***Yanagawa N, et al. J Thorac Oncol. 2012 Oct;7(10):1513-21.

Supplementary S2: Baseline characteristics of patients

Characteristic	Biomarker population (*N*=324)	Centers with samples (*N*=673)	Entire study population (*N*=760)
**Gender, n (%)**			
Male	223 (69%)	448 (67%)	504 (66%)
Female	101 (31%)	225 (33%)	256 (34%)
**Age, median (interquartile range)**	62 (57-67)	62 (56-67)	62 (56-66)
**Ethnicity, n (%)**			
Caucasian	303 (94%)	642 (95%)	727 (96%)
East Asian	17 (5%)	23 (3%)	24 (3%)
Other	4 (1%)	8 (1%)	9 (1%)
**Smoking status, n (%)**			
Never smoker	63 (19%)	128 (19%)	157 (21%)
Former smoker	178 (55%)	367 (55%)	404 (53%)
Current smoker	83 (26%)	178 (26%)	199 (26%)
**ECOG performance status, n (%)**			
0	156 (48%)	323 (48%)	382 (50%)
1	168 (52%)	350 (52%)	378 (50%)
**Stage, n (%)**			
IIIB	34 (10%)	71 (11%)	83 (11%)
IV	290 (90%)	602 (89%)	677 (89%)
**Histology, n (%)**			
Squamous,	61 (19%)	122 (18%)	141 (19%)
Large cell	15 (5%)	27 (4%)	28 (4%)
Mixed	11 (3%)	13 (2%)	13 (2%)
NSCLC not otherwise specified	56 (17%)	141 (21%)	156 (21%)
Adenocarcinoma	181 (56%)	370 (55%)	422 (56%)
**Treatment arm, n (%)**			
Standard arm	164 (51%)	337 (50%)	380 (50%)
Experimental arm	160 (49%)	336 (50%)	380 (50%)

Supplementary Table S3: Patients characteristics by EGFR mutational status

	***EGFR* mutational status (*N*=275)**		
	**wild type (*N*=236)**	**mutated (*N*=39)**	***P* value***
**Gender, n (%)**			0.0001
Male	175 (74%)	17 (44%)	
Female	61 (26%)	22 (56%)	
**Age, median (range)**	62 (26-77)	63 (44-75)	0.25
**Ethnicity, n (%)**			<0.0001
Caucasian	227 (96%)	28 (72%)	
East Asian	7 (3%)	10 (26%)	
Other	2 (1%)	1 (3%)	
**Smoking status, n (%)**			<0.0001
Never smoker	28 (12%)	25 (64%)	
Former smoker	138 (58%)	10 (26%)	
Current smoker	70 (30%)	4 (10%)	
**ECOG performance status, n (%)**			0.07
0	108 (46%)	24 (62%)	
1	128 (54%)	15 (38%)	
**Stage, n (%)**			0.38
IIIB	25 (11%)	6 (15%)	
IV	211 (89%)	33 (85%)	
**Histology, n (%) ** **			0.44
Squamous,	51 (22%)	4 (10%)	
Large cell	12 (5%)	1 (3%)	
Mixed	7 (3%)	2 (5%)	
NSCLC not otherwise specified	39 (17%)	7 (18%)	
Adenocarcinoma	127 (54%)	25 (64%)	

*Wilcoxon test for age, Chi square test in all other cases

** Histology as declared at baseline

Supplementary Table S4: Patients characteristics according to EGFR gene copy number

	EGFR gene copy number (*N*=196)		
	**FISH- (*N*=94)**	**FISH+ (*N*=102)**	***P* value***
**Gender, n (%)**			0.93
Male	64 (68%)	70 (69%)	
Female	30 (32%)	32 (31%)	
**Age, median (range)**	63 (26-77)	61 (38-77)	0.45
**Ethnicity, n (%)**			0.52
Caucasian	87 (93%)	92 (90%)	
East Asian	5 (5%)	9 (9%)	
Other	2 (2%)	1 (1%)	
**Smoking status, n (%)**			0.64
Never smoker	15 (16%)	20 (20%)	
Former smoker	55 (59%)	53 (52%)	
Current smoker	24 (26%)	29 (28%)	
**ECOG performance status, n (%)**			
0	49 (52%)	48 (47%)	0.48
1	45 (48%)	54 (53%)	
**Stage, n (%)**			
IIIB	13 (14%)	9 (9%)	0.27
IV	81 (86%)	93 (91%)	
**Histology, n (%) ****			
Squamous	21 (22%)	16 (16%)	
Large cell	6 (6%)	2 (2%)	0.31
Mixed	5 (5%)	4 (4%)	
NSCLC not otherwise specified	13 (14%)	18 (18%)	
Adenocarcinoma	49 (52%)	62 (61%)	

*Wilcoxon test for age, Chi square test in all other cases

** Histology as declared at baseline

Supplementary Table S5: Patients characteristics according to KRAS mutational status

	KRAS mutational status (*N*=276)		
	**wild type (*N*=203)**	**mutated (*N*=73)**	***P* value***
**Gender, n (%)**			
Male	145 (71%)	48 (66%)	0.36
Female	58 (29%)	25 (34%)	
**Age, median (range)**	62 (26-77)	68 (38-77)	0.59
**Ethnicity, n (%)**			
Caucasian	185 (91%)	70 (96%)	0.36
East Asian	15 (7%)	2 (3%)	
Other	3 (1%)	1 (1%)	
**Smoking status, n (%)**			
Never smoker	47 (23%)	5 (7%)	0.009
Former smoker	106 (52%)	46 (63%)	
Current smoker	50 (25%)	22 (30%)	
**ECOG performance status, n (%)**			
0	102 (50%)	31 (42%)	0.25
1	101 (50%)	42 (58%)	
**Stage, n (%)**			
IIIB	25 (12%)	5 (7%)	0.20
IV	178 (88%)	68 (93%)	
**Histology, n (%) ****			
Squamous	41 (20%)	14 (19%)	0.57
Large cell	12 (6%)	1 (1%)	
Mixed	7 (3%)	2 (3%)	
NSCLC not otherwise specified	32 (16%)	14 (19%)	
Adenocarcinoma	111 (55%)	42 (58%)	

*Wilcoxon test for age, Chi square test in all other cases

** Histology as declared at baseline

Supplementary Table S6: Patients characteristics according to EGFR IHC

	EGFR IHC score (*N*=164)		
	**EGFR IHC low (*N*=147)**	**EGFR IHC high (*N*=17)**	***P* value***
**Gender, n (%)**			
Male	105 (71%)	10 (59%)	0.28
Female	42 (29%)	7 (41%)	
**Age, median (range)**	61 (26-77)	58 (50-75)	0.86
**Ethnicity, n (%)**			
Caucasian	136 (93%)	16 (94%)	0.93
East Asian	10 (7%)	1 (6%)	
Other	1 (1%)	0	
**Smoking status, n (%)**			
Never smoker	24 (16%)	2 (12%)	0.74
Former smoker	81 (55%)	11 (65%)	
Current smoker	42 (29%)	4 (24%)	
**ECOG performance status, n (%)**			
0	73 (50%)	5 (29%)	0.11
1	74 (50%)	12 (71%)	
**Stage, n (%)**			
IIIB	19 (13%)	1 (6%)	0.40
IV	128 (87%)	16 (94%)	
**Histology, n (%) ****			
Squamous	25 (17%)	7 (41%)	0.13
Large cell	8 (5%)	0	
Mixed	8 (5%)	0	
NSCLC not otherwise specified	22 (15%)	3 (18%)	
Adenocarcinoma	84 (57%)	7 (41%)	

*Wilcoxon test for age, Chi square test in all other cases

** Histology as declared at baseline

Supplementary Table S7: Patients characteristics according to PTEN IHC

	PTEN IHC score (*N*=148)		
	**PTEN IHC negative (*N*=30)**	**PTEN IHC positive (*N*=118)**	***P* value***
**Gender, n (%)**			
Male	25 (83%)	78 (66%)	0.07
Female	5 (17%)	40 (34%)	
**Age, median (range)**	59 (41-71)	62 (26-77)	0.12
**Ethnicity, n (%)**			
Caucasian	29 (97%)	108 (92%)	0.61
East Asian	1 (3%)	9 (8%)	
Other	0	1 (1%)	
**Smoking status, n (%)**			
Never smoker	3 (10%)	20 (17%)	0.44
Former smoker	20 (67%)	64 (54%)	
Current smoker	7 (23%)	34 (29%)	
**ECOG performance status, n (%)**			
0	15 (50%)	54 (46%)	0.68
1	15 (50%)	64 (54%)	
**Stage, n (%)**			
IIIB	3 (10%)	15 (13%)	0.68
IV	27 (90%)	103 (87%)	
**Histology, n (%) ** **			
Squamous,	9 (30%)	19 (16%)	0.45
Large cell	1 (3%)	6 (5%)	
Mixed	1 (3%)	7 (6%)	
NSCLC not otherwise specified	3 (10%)	20 (17%)	
Adenocarcinoma	16 (53%)	66 (56%)	

*Wilcoxon test for age, Chi square test in all other cases

** Histology as declared at baseline

Supplementary Table S8: Patients characteristics according to c-MET IHC H-score (low vs high)

	c-MET IHC H-score (*N*=154)		
	**c-MET IHC H-score low (*N*=85)**	**c-MET IHC H-score high (*N*=69)**	***P* value***
**Gender, n (%)**			0.49
Male	61 (72%)	46 (67%)	
Female	24 (28%)	23 (33%)	
**Age, median (range)**	61 (39-77)	63 (26-77)	0.47
**Ethnicity, n (%)**			
Caucasian	78 (92%)	64 (93%)	0.66
East Asian	6 (7%)	5 (7%)	
Other	1 (1%)	0	
**Smoking status, n (%)**			
Never smoker	12 (14%)	14 (20%)	0.45
Former smoker	51 (60%)	35 (51%)	
Current smoker	22 (26%)	20 (29%)	
**ECOG performance status, n (%)**			0.84
0	38 (45%)	32 (46%)	
1	47 (55%)	37 (54%)	
**Stage, n (%)**			0.81
IIIB	10 (12%)	9 (13%)	
IV	75 (88%)	60 (87%)	
**Histology, n (%) ****			
Squamous	26 (31%)	4 (6%)	0.00009
Large cell	5 (6%)	2 (3%)	
Mixed	0	7 (10%)	
NSCLC not otherwise specified	10 (12%)	14 (20%)	
Adenocarcinoma	44 (52%)	42 (61%)	

*Wilcoxon test for age, Chi square test in all other cases

** Histology as declared at baseline

Supplementary Table S9: Patients characteristics according to c-MET IHC modified score

	c-MET IHC modified score (*N*=154)		
	**c-MET IHC negative (*N*=64)**	**c-MET IHC positive (*N*=90)**	***P* value***
**Gender, n (%)**			0.37
Male	47 (73%)	60 (67%)	
Female	17 (27%)	30 (33%)	
**Age, median (range)**	61 (39-74)	60 (26-77)	0.59
**Ethnicity, n (%)**			
Caucasian	59 (92%)	83 (92%)	0.47
East Asian	4 (6%)	7 (8%)	
Other	1 (2%)	0	
**Smoking status, n (%)**			
Never smoker	7 (11%)	19 (21%)	0.25
Former smoker	38 (59%)	48 (53%)	
Current smoker	19 (30%)	23 (26%)	
**ECOG performance status, n (%)**			0.53
0	31 (48%)	39 (43%)	
1	33 (52%)	51 (57%)	
**Stage, n (%)**			0.96
IIIB	8 (12%)	11 (12%)	
IV	56 (88%)	79 (88%)	
**Histology, n (%) ** **			
Squamous	21 (33%)	9 (10%)	0.0013
Large cell	4 (6%)	3 (3%)	
Mixed	0	7 (8%)	
NSCLC not otherwise specified	10 (16%)	14 (16%)	
Adenocarcinoma	29 (45%)	57 (63%)	

*Wilcoxon test for age, Chi square test in all other cases

** Histology as declared at baseline

Supplementary Table S10: Patients characteristics according to EGFR -216 genotype category

	EGFR -216 (*N*=240)		
	EGFR -216 G/G **(*N*=78)**	***EGFR -216 T/-*** **(*N*=162)**	***P*** **value***
**Gender, n (%)**			
Male	49 (63%)	112 (69%)	0.33
Female	29 (37%)	50 (31%)	
**Age, median (range)**	62 (40-77)	62 (26-77)	0.52
**Ethnicity, n (%)**			
Caucasian	71 (91%)	158 (98%)	0.056
East Asian	6 (8%)	4 (2%)	
Other	1 (1%)	0	
**Smoking status, n (%)**			
Never smoker	17 (22%)	30 (19%)	0.058
Former smoker	49 (63%)	84 (52%)	
Current smoker	12 (15%)	48 (30%)	
**ECOG performance status, n (%)**			
0	41 (53%)	77 (48%)	0.47
1	37 (47%)	85 (52%)	
**Stage, n (%)**			
IIIB	9 (12%)	16 (10%)	0.69
IV	69 (88%)	146 (90%)	
**Histology, n (%) ** **			
Squamous,	16 (21%)	33 (20%)	0.88
Large cell	4 (5%)	8 (5%)	
Mixed	1 (1%)	6 (4%)	
NSCLC not otherwise specified	13 (17%)	24 (15%)	
Adenocarcinoma	44 (56%)	91 (56%)	

*Wilcoxon test for age, Chi square test in all other cases

** Histology as declared at baseline

Supplementary Table S11: Patients characteristics according to EGFR -191 genotype category

	EGFR -191 (*N*=240)		
	***EGFR -191 C/C* (*N*=195)**	***EGFR -191 A/-* (*N*=45)**	***P* value***
**Gender, n (%)**			
Male	138 (71%)	23 (51%)	0.011
Female	57 (29%)	22 (49%)	
**Age, median (range)**	60 (41 – 77)	62 (26 – 77)	0.74
**Ethnicity, n (%)**			
Caucasian	185 (95%)	44 (98%)	0.036
East Asian	10 (5%)	0	
Other	0	1 (2%)	
**Smoking status, n (%)**			
Never smoker	39 (20%)	8 (18%)	0.36
Former smoker	104 (53%)	29 (64%)	
Current smoker	52 (27%)	8 (18%)	
**ECOG performance status, n (%)**			
0	95 (49%)	23 (51%)	0.77
1	100 (51%)	22 (49%)	
**Stage, n (%)**			
IIIB	21 (11%)	4 (9%)	0.71
IV	174 (89%)	41 (91%)	
**Histology, n (%) ****			
Squamous	41 (21%)	8 (18%)	0.42
Large cell	8 (4%)	4 (9%)	
Mixed	6 (3%)	1 (2%)	
NSCLC not otherwise specified	31 (16%)	6 (13%)	
Adenocarcinoma	109 (56%)	26 (58%)	

*Wilcoxon test for age, Chi square test in all other cases

** Histology as declared at baseline

Supplementary Table S12: Patients characteristics according to ABCG2 +421 polymophism

	***ABCG2* (*N*=257)**		
	***ABCG2 C/C* (*N*=218)**	***ABCG2 C/A or A/A* (*N*=39)**	***P* value***
**Gender, n (%)**			
Male	147 (67%)	24 (62%)	0.47
Female	71 (33%)	15 (38%)	
**Age, median (range)**	62 (26-77)	59 (34 – 71)	0.09
**Ethnicity, n (%)**			
Caucasian	211 (97%)	32 (82%)	0.0003
East Asian	6 (3%)	7 (18%)	
Other	1 (<1%)	0	
**Smoking status, n (%)**			
Never smoker	42 (19%)	11 (28%)	0.017
Former smoker	126 (58%)	13 (33%)	
Current smoker	50 (23%)	15 (38%)	
**ECOG performance status, n (%)**			
0	107 (49%)	20 (51%)	0.80
1	111 (51%)	19 (49%)	
**Stage, n (%)**			
IIIB	22 (10%)	5 (13%)	0.61
IV	196 (90%)	34 (87%)	
**Histology, n (%) ****			
Squamous,	43 (20%)	7 (18%)	0.67
Large cell	12 (6%)	1 (3%)	
Mixed	8 (4%)	0	
NSCLC not otherwise specified	36 (17%)	7 (18%)	
Adenocarcinoma	119 (55%)	24 (62%)	

*Wilcoxon test for age, Chi square test in all other cases

** Histology as declared at baseline

Supplementary Table S13: Patients characteristics according to EGFR CA dinucleotide repeat length genotypes

	***EGFR CA* repeat (*N*=262)**		
	***EGFR CA* Short/Short (*N*=74)**	**EGFR CA Long/- (*N*=188)**	***P* value***
**Gender, n (%)**			
Male	52 (70%)	123 (65%)	0.45
Female	22 (30%)	65 (35%)	
**Age, median (range)**	62 (26-73)	62 (38-77)	0.80
**Ethnicity, n (%)**			
Caucasian	71 (96%)	177 (94%)	0.75
East Asian	3 (4%)	10 (5%)	
Other	0	1 (1%)	
**Smoking status, n (%)**			
Never smoker	12 (16%)	41 (22%)	0.52
Former smoker	44 (59%)	99 (53%)	
Current smoker	18 (24%)	48 (26%)	
**ECOG performance status, n (%)**			
0	38 (51%)	90 (48%)	0.61
1	36 (49%)	98 (52%)	
**Stage, n (%)**			
IIIB	7 (9%)	20 (11%)	0.78
IV	67 (91%)	168 (89%)	
**Histology, n (%) ****			
Squamous,	19 (26%)	33 (18%)	0.55
Large cell	2 (3%)	11 (6%)	
Mixed	2 (3%)	6 (3%)	
NSCLC not otherwise specified	11 (15%)	32 (17%)	
Adenocarcinoma	40 (54%)	106 (56%)	

Short (S) is CA repeat ≤16; Long (L) is CA repeat >16; Patients are classified as S/S vs. L/- (L/S or L/L) groups.

*Wilcoxon test for age, Chi square test in all other cases

** Histology as declared at baseline

Supplementary Table S14: Relationships between biomarkers expressed as odds ratios

	EGFR mutation	EGFR gene copy	EGFR IHC	KRAS mutation	PTEN	cMET	cMET modified	EGFR SNP 216	EGFR SNP 191	ABCG2	EGFR CA repeat
**EGFR mutation**		6.13	1.51	0.06	1.37	1.73	1.87	1.88	0.85	0.90	1.11
**EGFR gene copy**	0.0013		1.89	1.32	1.33	0.77	1.31	0.64	0.86	0.62	1.32
**EGFR IHC**	0.55	0.24		1.10	1.24	0.65	1.03	1.46	0.17	2.86	0.62
**KRAS mutation**	0.007	0.39	0.86		1.22	1.22	0.98	2.74	0.51	1.02	0.94
**PTEN**	0.63	0.52	0.75	0.68		4.44	4.00	0.57	0.60	0.86	1.15
**cMET**	0.25	0.45	0.43	0.58	0.002		**417**	0.55	0.78	0.85	1.30
**cMET modified**	0.22	0.45	0.96	0.97	0.0014	**<0.0001**		0.52	1.21	0.80	1.01
EGFR -216	0.12	0.19	0.50	0.62	0.24	0.13	0.09		0.42	1.82	0.52
EGFR -191	0.74	0.72	0.003	0.08	0.44	0.59	0.69	0.01		0.50	4.78
ABCG2 +421	0.85	0.26	0.32	0.97	0.80	0.73	0.65	0.16	0.22		1.01
***EGFR CA* repeat**	0.80	0.45	0.48	0.85	0.77	0.51	0.98	0.05	0.004	0.98	

Genetic polymorphisms were analysed as dichotomous variables. Odds ratios (OR) were estimated for each pairwise association. Two triangular tables with OR (top right) and *P* values (bottom left) are provided, with significance level of association is pre-defined at 0.001. Unknown category was be considered.

Values above the diagonal represent Odds Ratios, to measure pairwise associations. Values under the diagonal represent the corresponding *P* values (in bold *P* values ≤0.001).

Supplementary Table S15: Skin toxicity and diarrhea according to polymorphisms

Polymorphism / Treatment Arm	Any skin toxicity: Genotypes		Odds ratio (95% CI)	*P* value (Zelen)	Any diarrhea: Genotypes		Odds ratio (95% CI)	*P* value (Zelen)
EGFR -216	***G/G***	***G/T or T/T***			***G/G***	***G/T or T/T***		
Cisplatin/Gemcitabine	6/37 (16%)	10/78 (13%)	1.32 (0.44-3.94)	>0.99	6/37 (16%)	6/78 (8%)	2.32 (0.69-7.77)	0.71
Erlotinib	32/40 (80%)	60/83 (72%)	1.53 (0.62-3.82)		18/40 (45x%)	30/83 (36%)	1.46 (0.67-3.11)	
EGFR -191	***C/C***	***C/A or A/A***			***C/C***	***C/A or A/A***		
Cisplatin/Gemcitabine	11/93 (12%)	5/22 (23%)	0.46 (0.14-1.48)	>0.99	11/93 (12%)	1/22 (5%)	2.82 (0.34-23.06)	0.37
Erlotinib	72/100 (72%)	20/23 (87%)	0.39 (0.11-1.40)		37/100 (37%)	11/23 (48%)	0.64 (0.26-1.60)	
ABCG2 +421	***C/C***	***C/A or A/A***			***C/C***	***C/A or A/A***		
Cisplatin/Gemcitabine	13/103 (13%)	3/22 (14%)	0.91 (0.24-3.53)	0.65	12/103 (12%)	2/22 (9%)	1.32 (0.27-6.36)	>0.99
Erlotinib	82/114 (72%)	15/17 (88%)	0.34 (0.07-1.58)		44/114 (39%)	7/17 (41%)	0.90 (0.32-2.53)	
***EGFR CA* repeat**	***S/S***	***S/L or L/L***			***S/S***	***S/L or L/L***		
Cisplatin/Gemcitabine	1/22 (5%)	15/104 (14%)	0.28 (0.04-2.26)	0.41	2/22 (9%)	12/104 (12%)	0.77 (0.16-3.70)	>0.99
Erlotinib	28/38 (74%)	72/96 (75%)	0.93 (0.40-2.20)		15/38 (39%)	39/96 (41%)	0.95 (0.44-2.05)	

CI: confidence interval

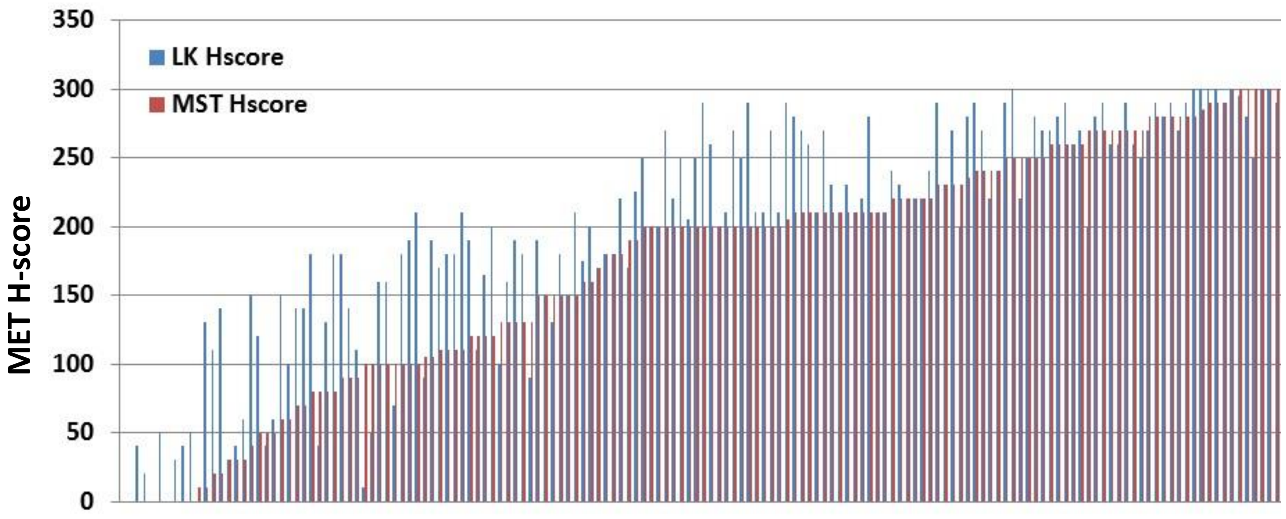

Figure S1. Comparison of the 2 independent cMET H-scores.

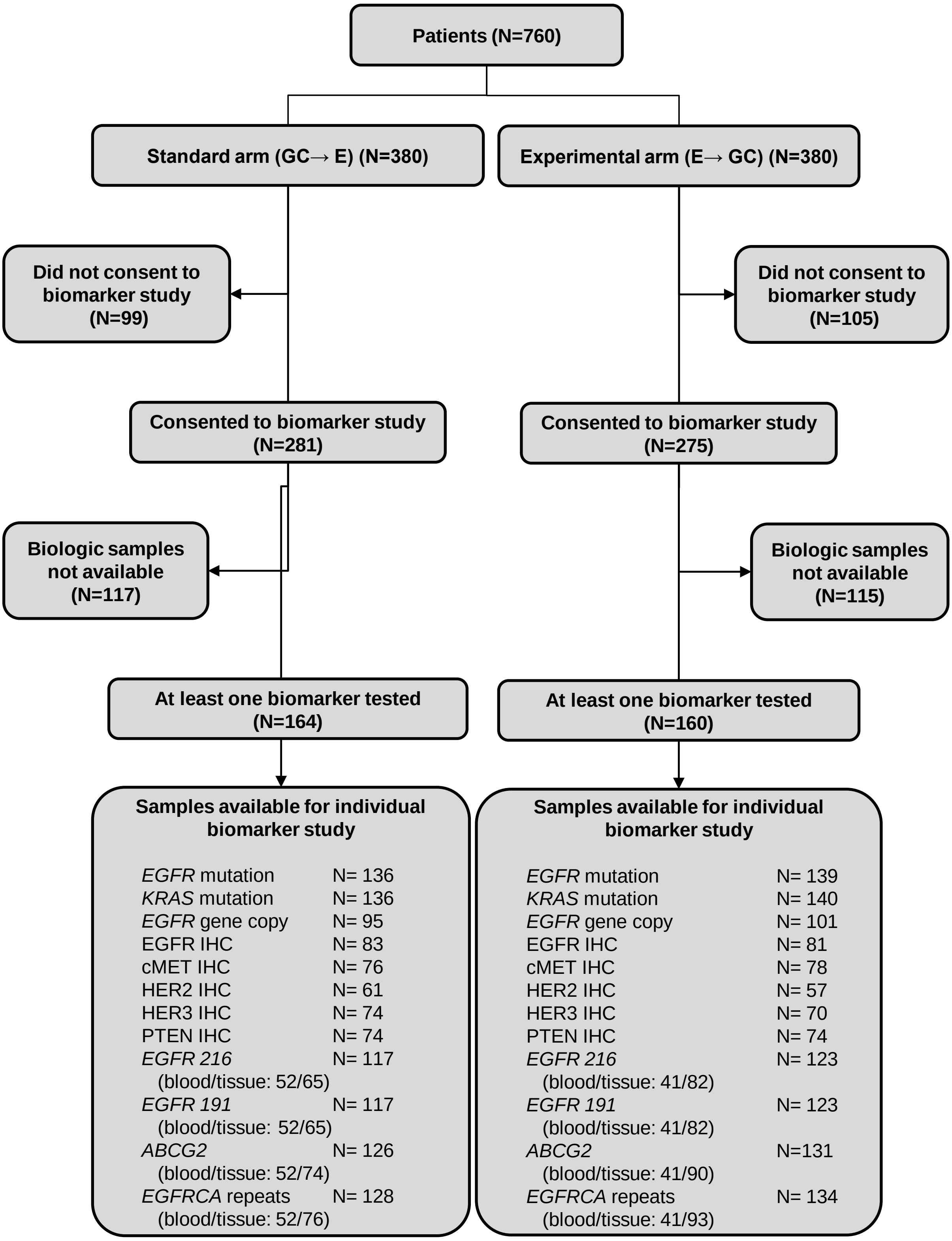
Patients (N=760)
Standard arm (GC→ E) (N=380)
Experimental arm (E→ GC) (N=380)
Did not consent to biomarker study (N=99)
Did not consent to biomarker study (N=105)
Consented to biomarker study (N=281)
Consented to biomarker study (N=275)
Biologic samples not available (N=117)
Biologic samples not available (N=115)
At least one biomarker tested (N=164)
At least one biomarker tested (N=160)
Samples available for individual biomarker study
EGFR mutation N= 136
KRAS mutation N= 136
EGFR gene copy N= 95
EGFR IHC N= 83
cMET IHC N= 76
HER2 IHC N= 61
HER3 IHC N= 74
PTEN IHC N= 74
EGFR 216 N= 117
(blood/tissue: 52/65)
EGFR 191 N= 117
(blood/tissue: 52/65)
ABCG2 N= 126
(blood/tissue: 52/74)
EGFRCA repeats N= 128
(blood/tissue: 52/76)
Samples available for individual biomarker study
EGFR mutation N= 139
KRAS mutation N= 140
EGFR gene copy N= 101
EGFR IHC N= 81
cMET IHC N= 78
HER2 IHC N= 57
HER3 IHC N= 70
PTEN IHC N= 74
EGFR 216 N= 123
(blood/tissue: 41/82)
EGFR 191 N= 123
(blood/tissue: 41/82)
ABCG2 N=131
(blood/tissue: 41/90)
EGFRCA repeats N= 134
(blood/tissue: 41/93)

Supplementary Figure S2: Patient study flow and conduction of biomarker study by treatment arm

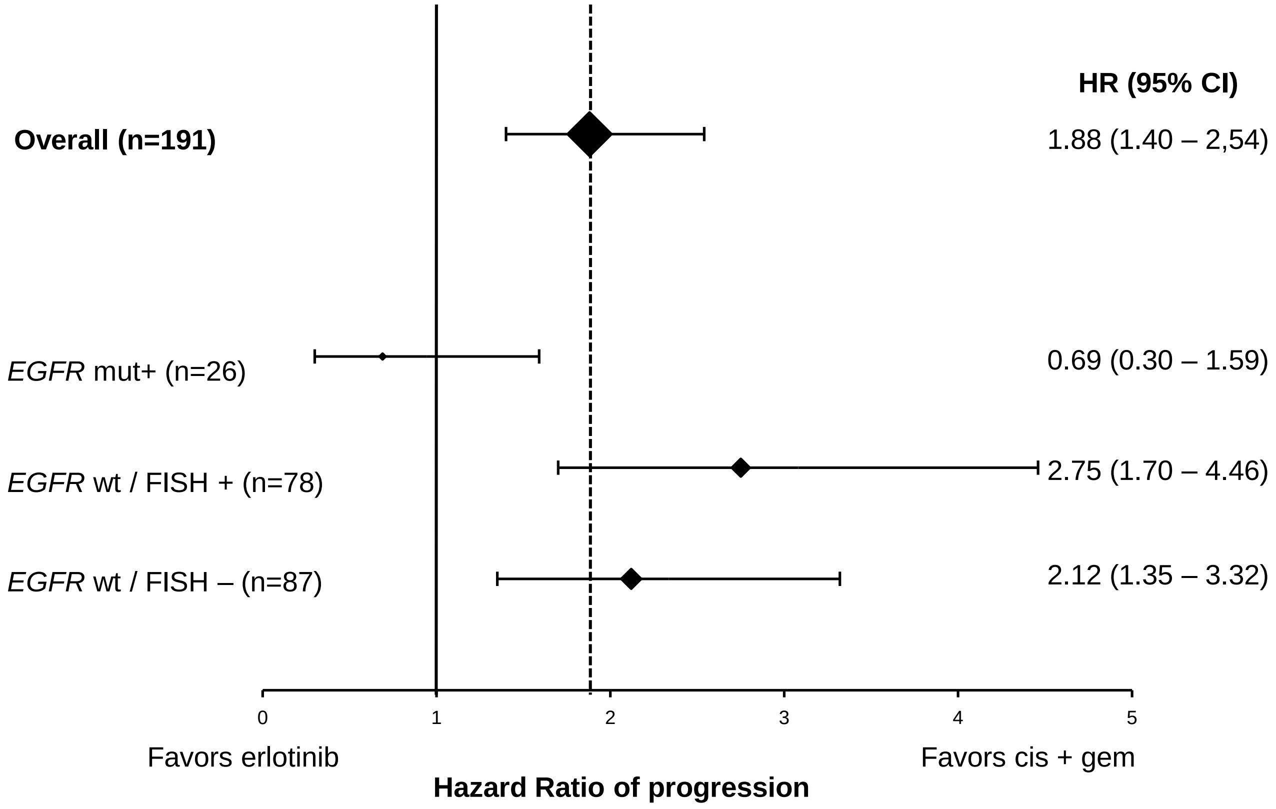

Supplementary Figure S3: Forest plot of HR of progression by *EGFR* gene copy number in *EGFR* wild type patients

TORCH Investigators

Steering Committee

Cesare Gridelli, Charles Butts, Fortunato Ciardiello, Ronald Feld, Ciro Gallo, Francesco Perrone.

Study coordination

Italy: Francesco Perrone (study coordinator), Alessandro Morabito, Massimo Di Maio, Maria Carmela Piccirillo, Gennaro Daniele, Ermelinda De Maio[a], Jane Bryce, Manuela Florio, Federika Crudele, Antonia Del Giudice, Alfonso Savio, Gianfranco De Feo, Fabiano Falasconi, Roberta D'Aniello, Maria Rosaria Salzano, Aldo Vecchione (National Cancer Institute, Napoli).

Canada: Ronald Feld (study coordinator), Jessie Miller, Jackie Amaral, Sandra Harb, Pamela Degendorfer (Princess Margaret Hospital, Toronto).

Statistical analysis

Ciro Gallo, Paolo Chiodini, Simona Signoriello, Giuseppe Signoriello (Second University, Napoli, Italy).

Investigators

Italy: Vittorio Gebbia, Gianfranco Mancuso, Antonio Testa, Eugenio Bajardi, Carlo Arcara (Casa di Cura La Maddalena, Palermo); Cesare Gridelli, Paolo Maione, Antonio Rossi, Clorinda Schettino, Marianna Bareschino (S.G.Moscati Hspital, Avellino); Fortunato Ciardiello, Floriana Morgillo, Flavia Cantile; Ferdinando De Vita, Morena Fasano, Erika Martinelli, Michele Orditura, Teresa Troiani, Ciro Gallo, Simona Signoriello, Paolo Chiodini, Giuseppe Signoriello (Second University, Napoli); Giovenzio Genestreti, Manuela Monti, Carlo Milandri, Ruggero Ridolfi (Istituto Scientifico Romagnolo per lo Studio e la Cura dei Tumori, Meldola [FO]); Adolfo Favaretto, Giulia Pasello, Laura Bonanno, Paolo Carli (Istituto Oncologico Veneto, Padova); Saverio Cinieri, Angelo Nacci, Pietro Rizzo, Palma Fedele (A.Perrino Hospital, Brindisi and European Institute of Oncology, Milano); Salvatore Siena, Mauro Moroni, Giovanna Marrapese, Giulio Cerea (Niguarda Ca' Granda Hospital, Milano); Giampaolo Tortora[b], Roberto Bianco, Roberta Marciano, Elide Matano (Federico II University, Napoli); Raffaella Felletti, Mercedes Paquali , Giorgio Bernabò (San Martino Hospital, Genova); Ferdinando Riccardi, Giacomo Cartenì, Chiara De Vitiis, Mimma Rizzo (Cardarelli Hospital, Napoli), Mario Spatafora, Gaetana Camarda, Vincenzo Bellia (V.Cervello Hospital, Palermo); Anna Ceribelli, Francesco Cognetti, Maria Tedeschi (Regina Elena Institute, Roma); Enzo Pasquini, Davide Tassinari, Maximilian Papi (Ospedale degli Infermi, Cattolica/Civil Hospital, Rimini); Vittorio Fregoni, Anna Milani, Lorenzo Pavesi (Salvatore Maugeri Foundation, Pavia); Luigi Cavanna, Carmelina Rodinò, Elena Zaffignani (Guglielmo da Saliceto Hospital, Piacenza); Manlio Mencoboni, Andrea Burzone, Maria Grazia Covesnon (Villa Scassi Hospital, Genova Sampierdarena); Bruno Daniele, Emiddio Barletta, Vincenza Tinessa (G. Rummo Hospital, Benevento); Francesco Rosetti, Orazio Vinante, Giovanni Luigi Papagallo (U.L.S.S. 13, Mirano [VE]); Giuseppe Valmadre, Renzo Epis (E.Morelli Hospital, Sondalo); Fabrizio Artioli, Ilaria Bernardini (Ramazzini Hospital, Carpi [MO]), Vincenzo Adamo, Tindara Franchina (G.Martino University, Messina); Giuditta D'Isernia, Marco Ciaparrone (S. Giovanni Calibita

Fatebenefratelli Hospital, Roma); Alfonso Maria D'Arco, Annamaria Libroia (Umberto I Civil Hospital, Nocera Inferiore [SA]); Enzo Veltri, Maria Colonna (Gaeta Hospital, Gaeta [LT]); Alessandra Bearz, Umberto Tirelli (C.R.O., Aviano [PN]); Francesco Carrozza, Michela Musacchio (A.Cardarelli Hospital, Campobasso); Santi Barbera, Francesco Renda (Mariano Santo Hospital, Cosenza); Michele Maio, Luana Calabrò (AO Universitaria Policlinico Le Scotte, Siena); Elena Piazza, Virginio Filipazzi (L.Sacco Hospital, Milano); Claudio Verusio, Raffaella Morena (Busto Arsizio Hospital, Saronno); Davide Santeufemia, Alessandro Del Conte (S.Maria degli Angeli Hospital, Pordenone); Daniela Pozzessere, Francesca Del Monte (Prato Hospital, Prato); Teresa Gamucci (Umberto I Hospital, Frosinone); Vito Barbieri (Magna Grecia University - Mater Domini Hospital, Catanzaro); Stefano Tamberi (Civil Hospital Faenza, Faenza [RA]); Elisa Varriale (Buon Consiglio Fatebenefratelli Hospital, Napoli); Rodolfo Mattioli (S. Croce Hospital, Fano [PS]); Giovanna Antonelli (S. Vincenzo Hospital, Taormina [ME]); Enrico Aitini (C. Poma Hospital, Mantova); David Rossi (S. Salvatore Hospital, Pesaro); Francesco Testore (Cardinal Massaia Hospital, Asti); Edmondo Terzoli (Regina Elena Institute, Roma); Modesto D'Aprile (S.Maria Goretti Hospital, Latina); Domenico Galetta (Istituto Oncologico di Bari, Bari); Antonio Ghidini (Casa di Cura IGEA, Milano); Elvira De Marino (S.Andrea Hospital, Vercelli); Luciana Irtelli (G.D'Annunzio - Chieti University, Chieti); Elena Raisi (Arcispedale Sant'Anna, University, Ferrara); Luca Moscetti (Belcolle Hospital, Viterbo); Maria Rosaria Valerio (Giaccone University, Palermo); Michele Caruso (Humanitas Centro Catanese di Oncologia, Catania); Pietro Masullo (S.Luca Hospital, Vallo della Lucania [SA]); Vincenzo Chiuri (Vito Fazzi Hospital, Lecce); Sergio Montanara (ASL14, Verbania); Francesco Perrone, Alessandro Morabito, Massimo Di Maio, Maria Carmela Piccirillo, Gennaro Daniele, Ermelinda De Maio[a], Raffaele Costanzo, Jane Bryce, Roberta D'Aniello, Maria Rosaria Salzano, Gaetano Rocco, Aldo Vecchione (National Cancer Institute, Napoli).

Canada: Ronald Feld, Natasha Leighl, Ming-Sound Tsao, Mauro Saieg, Gilda da Cunha Santos, Ni Liu, Christine To, Olga Ludkovski, (Princess Margaret Hospital, Toronto); Rafal Wierzbicki (Durham Regional Cancer Centre, Oshawa); Zeenat Yasmin Alam, Sindu Kanjeekal, Tarek Elfiki, Swati Kulkarni (Windsor Regional Cancer Centre, Windsor); Victor Cohen, Jason Agulnik (Jewish General Hospital - McGill University Ca, Montreal); Brian Higgins, Robert Myers, Mark Rother (Credit Valley Hospital, Mississauga ON); Richard Gregg, Anna Tomiak, Mihaela Mates (Cancer Centre of Southeastern Ontario at KGH, Kingston); Quincy Chu, Anil Joy, David Fenton, Michael Smylie, Randeep Sangha (Cross Cancer Institute, Edmonton); Gary Harding, Srisala Navartnam (Cancer Care Manitoba, Winnipeg); John Goffin, Andrew Arnold (Juravinski Cancer Centre-St. Joseph's Hospital, Hamilton); Donald Morris, Desiree Hao (Tom Baker Cancer Centre, Calgary); Jonathan Noble (Hospital Regional de Sudbury Regional Hospital, Sudbury); Ronald Burkes (Mount Sinai Hospital, Toronto); Wojciech Morzycki (QEII Health Sciences Centre, Halifax); Cheryl Ho (BC Cancer Agency - Vancouver Centre, Vancouver).

Present address:

[a] Ospedali Riuniti - Azienda USL 6. Livorno

[b] University Hospital, Verona

Data managers

Italy: Manuela Florio, Federika Crudele, Giuliana Canzanella, Fiorella Romano, Giovanni de Matteis (Napoli); Paolo Russo (Palermo); Valentina Barbato, Rita Ambrosio (Avellino); Margherita Cinefra (Brindisi); Yves Franzosi (Milano); Ilaria Carbone (Roma); Giuliana Drudi , Barbara Venturini (Rimini); Camilla di Nunzio (Piacenza); Stefania Competiello (Benevento), Giorgia Razzini (Carpi- MO); Giovanni Amato (Siena); Barbara Barco, Alice Ballerio (Saronno); Antonino Ius (Pordenone); Filomena Narducci (Frosinone).

Canada: Jansen Janice (Toronto); Patricia Dupis (Windsor); Ricard Ginette (Montreal); Sandy Phillips (Mississauga ON); Christine Maize, Jackie Edwards (Kingston); Mary-Linn Gantefoer (Edmonton); Sandra Yap (Winnipeg); Moelker Yvonne (Hamilton); Kim Gerat (Calgary; Deb Bertrand (Sudbury); Janet Smith (Toronto).

Research nurses

Italy: Jane Bryce (Napoli); Noemi Giovannini (Forlì); Cristina Magro (Padova); Liana Letizia Falcone (Brindisi); Monica Mina (Milano); Antonio Messina (Genova); Silvia Coccato (Mirano -VE); Sergio Speranza (Siena); Maria Immacolata Morrone (Saronno); Manuela Gardonio (Pordenone).

Canada: Andrea Foster (Toronto); Cathy Pelham (Oshawa); Donna Fawdry (Windsor); Robin Rashcovsky (Montreal); Shelina Alarakhia (Mississauga ON); Carole Gallagher (Edmonton); Kathi Klapp (Winnipeg); Theresa Holmes (Hamilton); Sharon Holowachuk (Calgary); Faye Gee (Sudbury).

Biomarker studies

Lucia Kim, Mauro Saieg, Olga Ludkovski, Ni Liu, Chrystal Johnson, Christine To, James Ho, Nhu-An Pham, Zhuo Chen, Dangxiao Cheng, Devalben Patel, Geoffrey Liu, Ming-Sound Tsao (Toronto).
